# Social and spatial inequalities in healthcare use among people living with dementia in England (2002–2016)

**DOI:** 10.1080/13607863.2022.2107176

**Published:** 2022-08-12

**Authors:** James Watson, Mark A. Green, Clarissa Giebel, Frances Darlington-Pollock, Asangaedem Akpan

**Affiliations:** a School of Environmental Sciences, The University of Liverpool, Liverpool, United Kingdom; b Department of Primary Care and Mental Health, University of Liverpool, Liverpool, United Kingdom; c NIHR ARC NWC, Liverpool, United Kingdom; d Department of Sciences, University of York, York, United Kingdom; e Department of Medicine for Older People and Stroke, Liverpool University Hospitals NHS FT, Liverpool, United Kingdom; f Healthy Ageing Group, University of Cumbria, Cumbria, United Kingdom; g Institute of Life Course and Medical Sciences, University of Liverpool, Liverpool, United Kingdom; h NIHR CRN NWC, Liverpool, United Kingdom

**Keywords:** Dementia, primary healthcare, secondary healthcare, inequalities, socio-economic, spatial, routine data

## Abstract

**Objectives:**

Healthcare services for people living with dementia (PLWD) are stretched, and government promises of increased funding remain undelivered. With the UK dementia population to surpass 1 million by 2024, and dementia care costs predicted to almost treble by 2040, it is essential we understand differences in healthcare use among PLWD. This study aimed to explore social and spatial variations in healthcare use among people diagnosed with dementia (2002–2016).

**Methods:**

Data were derived from Electronic Health Records of Clinical Practice Research Datalink GP patients in England (*n* = 142,302). To standardise healthcare contacts, rates of healthcare contacts per year were calculated for three primary (GP observations and medications) and three secondary healthcare types [Accident & Emergency (A&E) attendances and, emergency and elective hospital admissions]. Fully-adjusted generalised linear regression models were used to identify healthcare use variation by social and spatial groups. Twelve models were generated, one for each healthcare type in early- and late-onset populations separately.

**Results:**

This study highlights numerous social and spatial variations in healthcare use among PLWD. Among PLWD, several groups tended to have healthcare service use more closely associated with negative outcomes, including a greater likelihood of A&E attendances and emergency and elective hospital admissions. These groups include: men, people from White ethnicity groups and people from more deprived and rural areas.

**Conclusions:**

Systemic and social measures are needed to reduce variations in healthcare use inequalities in PWLD. These include greater healthcare continuity, health checks and medicines reviews, culturally appropriate services, better and more accessible treatment and improved infrastructure.

## Introduction

Among people living with dementia (PLWD), inequalities exist in the availability and quality of healthcare (Cooper et al., 2017; Wu et al., 2018) and in the likelihood of negative health and social outcomes (Korhonen et al., 2020; van de Vorst et al., 2016; Watson et al., 2020). PLWD from disadvantaged areas and socio-economic groups experience greater unmet care needs, and have poorer health outcomes (Giebel et al., 2021; Wu et al., 2018). Recent government policy has prioritised reducing inequalities in accessing dementia diagnosis, support, treatment and resultant outcomes. However, commitments to increased funding to support services remain unfulfilled (Department of Health and Social Care, 2016; Local Government Association, 2021). Both health and social care are vital for PLWD and their carers to live well in the community or in a care home after a diagnosis, and continued lack of funding of both, and neglect of the social care system (King’s Fund, 2018), has resulted in an increased use of avoidable healthcare services (Alzheimer’s Society, 2018; National Institute for Health and Care Excellence (NICE), 2018).

The majority of PLWD are aged 65 years and over, and are more likely to have comorbidities than the general population (Griffith et al., 2016). The number of PLWD in the UK is expected to increase from an estimated 920,000 currently to over 1 million by 2024 (Wittenberg et al., 2019). The greatest increase will be among those with severe dementia symptomatology, with acute everyday support needs (Bennett et al., 2018). With increased and more acute need among PLWD, the cost of providing health and social care to PLWD is set to almost than treble by 2040 (Wittenberg et al., 2019). Increasing numbers of PLWD and more acute need, alongside sustained funding shortfalls will likely exacerbate inequalities in the accessibility and quality of healthcare, health outcomes and the frequency and cost of avoidable healthcare use.

Avoidable, unplanned healthcare use, including Accident & Emergency (A&E) attendances, hospital admissions and readmissions, is greater among PLWD than the general population (Voss et al., 2017). Among PLWD, there are differences in the likelihood of using potentially avoidable healthcare, by socio-economic and demographic groups, including by gender, age, levels of deprivation and rurality (Husaini et al., 2015; Thorpe et al., 2010; Shepherd et al., 2019; Watson et al., 2020). There are also social and spatial differences in the use of primary healthcare among PLWD, including the quality and frequency of dementia medications and, adequate care and treatment reviews (Cooper et al., 2017; Giebel et al., 2021; Lu et al., 2021). Avoidable healthcare use is associated with more severe dementia, faster deterioration, poorer quality of life, increased mortality risk and greater cost to the healthcare system (Briggs et al., 2017; Reynish et al., 2017; Sager et al., 1996; Tropea et al., 2017; van de Vorst et al., 2015). Although early diagnosis and effective treatment can reduce avoidable healthcare use and associated negative outcomes (Alzheimer’s Society, 2021; Watson et al., 2021), a lack of funding for formal services and greater and more acute need among PLWD will likely exacerbate avoidable healthcare use, leading to more proliferate negative health outcomes for PLWD with elevated costs to healthcare services. Some socio-economic groups and geographic areas are more likely to experience a lack of sufficient care, including those from more remote or historically underserved communities (Rahman et al., 2020; Thorpe et al., 2010; Watson et al., 2020). Funding issues, increased numbers of PLWD and more acute need is likely to widen existing inequalities, meaning those already experiencing poorer care, treatment and health outcomes will be affected more greatly.

It is therefore essential we understand the spatial and social contexts that influence the healthcare experiences of PLWD, to identify and address their resulting inequalities (Pearce et al., 2015). We define inequality here to mean observable differences between societal groups. We are describing the extent of these differences, and therefore we do not take an equity approach, however, inequalities often reflect unjust and unfair processes that lead to certain social groups to have better health than others. While some argue that inequalities reflect differences in need, these differences in need are often socially rooted as well. In our article, we select social and spatial factors that have been identified by the UK Government as unjust and use them as social markers for measuring inequalities. Providing a picture of differential need and quality and, avoidable service use based on spatial factors, can help with policy decisions to reduce pressure and financial burden on services and potentially address improved well-being for people with dementia (Dummer, 2008; Rice and Smith, 2001).

To reduce current and future inequalities among PLWD, we need to support better service delivery and healthcare decision-making. Electronic Healthcare Records (EHRs) can be used to identify healthcare use among large cohorts of patients with a specified health condition, such as dementia (Casey et al., 2016). EHRs have been employed previously to evidence inequalities in health outcomes (Watson et al., 2020, 2021). Understanding which services PLWD are in contact with, by social and spatial variables, can demonstrate differences associated with healthcare utilisation. There is a dearth of research evidencing contact with a multitude of healthcare services, or incorporating multiple explanatory factors of differences in healthcare use (Watson et al., 2020). Also, we are not aware of previous research exploring spatial variations in healthcare use among PLWD.

The aim of this study was to examine the extent to which social and spatial factors are associated with variations in the use of different types of primary and secondary healthcare among PLWD, using large-scale, longitudinal EHRs.

## Materials and methods

### Data access and ethical approval

Clinical Practice Research Datalink (CPRD) collect pseudo-anonymised, EHR from General Practices (GP) across the United Kingdom (UK). CPRD data incorporate ∼16 million patients registered with UK GPs representing 25% of the UK patient population. CPRD Aurum contains routinely-collected, anonymised EHR from registered GPs, covering primary care data, including GP contacts and medications. CPRD can also provide data linkage between primary and secondary healthcare records, social and spatial variables (CPRD, 2021). Data access was granted by CPRD and use of CPRD Aurum was approved by the University of Liverpool Research Ethics board (Reference: 7922).

### Sample population

**Figure 1.**
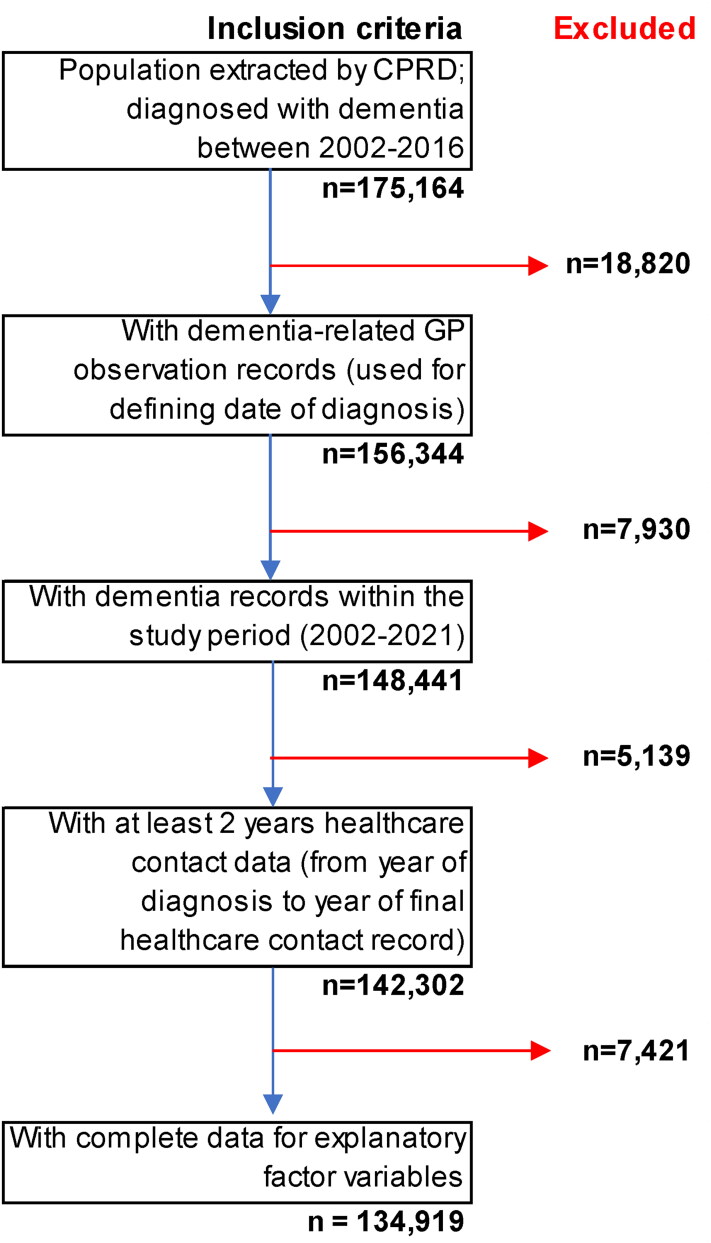
Inclusion/Exclusion criteria for sample population.

Patients registered with CPRD GPs, who were diagnosed with dementia between 2002 and 2016, with at least two years of follow-up healthcare data from date of diagnosis (Figure 1). Our initial analytical sample size was 142,302 people.

### Outcome variables

No date of dementia diagnosis is available in CPRD GP data. Dementia-specific GP observations are those that include one of the following terms as the reason the patient presented to their GP: ‘dementia’, ‘Alzheimer’, ‘cogniti’, or ‘memory’. We calculated date of diagnosis as the date for a patient’s first dementia-specific GP observation record occurred. Healthcare contacts included in analyses are only those that occurred after this diagnosis date.

This study includes six outcome variables distinguishing between different healthcare types. This includes three within primary healthcare (GP observations, dementia and non-dementia medications) and three within secondary healthcare (A&E attendances, emergency hospital admission spells and elective hospital admission spells). In this study, a healthcare contact refers to an individual record of communication or treatment between a PLWD and a healthcare service. Healthcare contacts were standardised for each member of the sample population. Rates for each of the six healthcare types were calculated, per year, based on years present in the data (from diagnosis to final record/date of death).

GP observations are self-contained, with one record for each observation at a GP visit. Dementia-specific medications include prescriptions for four drugs advised for use by the NHS for PLWD: Donepezil, Galantamine, Rivastigmine and Memantine. Non-dementia medications refer to all remaining drugs prescribed.

A&E attendance records are self-contained, denoting individual records of a person presenting at an Accident & Emergency department. Emergency hospital admission spells are records of urgent care need and elective hospital admission spells are records of planned care. A&E attendances are generally unplanned presentations at A&E or urgent care, and hospital admissions involve a clinical decision to admit the patient as they are deemed to require further care, treatment and observation.

### Explanatory variables

This study encompasses multiple variables as potential explanatory factors of variation in healthcare use among PLWD. Available from CPRD GP data, we included patients’ age at diagnosis, sex and GP region, and from patient secondary healthcare records, ethnicity. From age at diagnosis, we defined whether patients had early-onset (aged under 65 years) or late-onset dementia (aged 65 + years). People with early-onset dementia are more likely to have rarer forms of dementia than in late-onset (DementiaUK, 2022; Gupta et al., 2009), which can present additional symptomatology (Giebel et al., 2020). Together with the need for greater support with day-to-day activities, such as washing or preparing food, rare dementias can present varied symptoms that can have a greater impact on health and cognition (Gerritsen et al., 2019; Koedam et al., 2010; Smits et al., 2015). The differential impact on cognition and physical capabilities, along with family, social and employment dynamics mean people with early- and late-onset dementia will likely have differing needs (Alzheimer’s Society, 2020). 2015 Indices of Multiple Deprivation (IMD) quintile and GP urban/rural classification was available via data linkage using patients’ GP ID. This study includes these explanatory factors for healthcare use among PLWD, as research illustrates differential provision and quality of healthcare, and health outcomes for PLWD by age (continuous), sex, ethnicity and deprivation, and by spatial factors including level of urbanity/rurality (Rahman et al., 2020; Watson et al., 2020, 2021; Wu et al., 2020).

### Missing data

Our analytical sample size was 142,340 people. However, ethnicity data for 7,421 (5.2%) and IMD 2015 quintile data for 276 (0.2%) was missing data. As such these individuals were not included in regression analyses, with data assumed missing completely at random (Figure 1).

### Statistical analysis

The sample population was stratified into two groups based on age of onset of dementia diagnosis. Descriptive statistics of the sample populations’ social and spatial factors were calculated. Frequency counts and rates per year of the six healthcare types were calculated. Explanatory factors were included in fully-adjusted, generalised linear regression models, highlighting variation in healthcare use. A mixture of Binomial and Poisson generalised linear regression models were used. Those healthcare types with sufficient numbers of contacts were analysed using Poisson regression, based on rates per patient year. Those with insufficient numbers were based on binomial regression, based on whether the person did or did not use the type of healthcare. Within regression models, explanatory factors were included as dependent variables, with the rates/occurrence of healthcare contacts the independent variable(s).

*Early-onset*: Binomial regression models were used for dementia medications, A&E attendances and, elective and emergency hospital admissions. Poisson regression models were used for GP observations and non-dementia medications.

*Late-onset*: Binomial regression models were used for A&E attendances and, elective and emergency hospital admissions. Poisson regression models were used for GP observations, dementia and, non-dementia medications.

Early- and later-onset populations were analysed separately, with a total of 12 fully-adjusted models run to indicate differential use of each healthcare type by explanatory variables. Analyses were conducted in R. Poisson regression models return Incidence Rate Ratios (IIR), and Binomial regression models return odds ratios (OR), both with 95% confidence intervals. OR give us the relative difference to the reference group in the odds of an outcome, whereas IRR provide a ratio of the difference in the rate of the outcome compared to the reference group.

For categorical variables included as explanatory factors of an outcome in regression analyses, we are required to specify a level as our reference group, against which each of the other levels are compared. As a continuous variable in both early- and late-onset models, age at diagnosis did not require this. However, in our analyses, our reference groups for gender (women), ethnicity (White), urban-rural GP classification (Urban) are based on the level with the largest population size. For IMD 2015 deprivation quintile (Ministry of Housing, Communities & Local Government, 2015), we used the least deprived quintile (Quintile 5) as our reference group, to demonstrate the impact of increasing levels of deprivation on outcomes. For GP region, the North East was chosen as our reference group. In our descriptive analysis the North East was shown to have higher rates per year of most healthcare types than other regions, and so gave the most pragmatic choice for reference group.

## Results

### Sample population

Of the 142,302 population of PLWD (Table 1), approximately two-thirds were female, less than 4% were of Asian, Black or Mixed/Other ethnicity groups, and a greater proportion resided in less deprived areas. Less than 4% of the sample population had early-onset dementia, with the majority (78.9%) aged between 75 and 94 years. Approximately 1 in 7 was registered with GPs in urban areas and greater numbers were registered with GPs in the North West, West Midlands, South West and South-Central regions. Thirty-three people with dementia had neither IMD quintile, or ethnicity (<0.01%) available, 7,388 (5.2%) had no stated ethnicity and a further 243 (<0.2%) had no IMD quintile stated. Data are assumed missing at random, and not included in regression models.

**Table 1.** Demographic characteristics of sample population vs. UK dementia population.

Explanatory factors	Sample		UK^a^
	*n*	%	%
**Onset/Age group**			
Early-Onset	5,210	3.7%	5.2%
Under 45	104	0.1%	0.2%
45–54	870	0.6%	0.5%
55–64	4,236	3.0%	4.5%
Late-Onset	137,092	96.3%	94.8%
65.74	20,514	14.4%	16.6%
75.84	63,225	44.4%	36.5%
85–94	49,067	34.5%	36.2%
95+	4,286	3.0%	5.5%
**Sex**			
Female	94,033	66.1%	65.0%
Male	48,269	33.9%	35.0%
**Ethnicity**			
Asian^b^	1,937	1.4%	1.5%
Black^c^	2,246	1.6%	2.7%
Mixed/Other	1,080	0.8%	1.3%
White	129,618	91.1%	94.5%
**IMD 2015 deprivation Quintile**			
Quintile 1 (Most deprived)	22,362	15.7%	10.7%
Quintile 2	24,923	17.5%	16.6%
Quintile 3	28,601	20.1%	20.5%
Quintile 4	32,278	23.0%	23.4%
Quintile 5 (Least deprived)	33,362	23.4%	26.5%
**Urban–rural GP classification**			
Urban	121,586	85.4%	*NA*
Rural	20,719	14.6%	*NA*
**GP region** ^d^			
North East	7,428	5.2%	5.3%
North West	25,422	17.9%	13.5%
Yorkshire and The Humber	6,137	4.3%	9.9%
East Midlands	3,020	2.1%	9.3%
East of England	8,261	5.8%	11.8%
West Midlands	24,769	17.4%	10.2%
London	14,825	10.4%	11.0%
South Central	19,579	13.8%	*NA*
South East Coast	12,052	8.5%	17.3%
South West	20,809	14.6%	11.7%

a Dementia Statistics Hub, Alzheimer’s Research UK (May 2020).

b Sum of: Bangladeshi, Chinese, Indian, Other Asian, Pakistani ethnicities.

c Sum of: Black African, Black Caribbean, Black Other ethnicities.

d PHE Regions include only South East/South West.

Inclusion in the study required a date of diagnosis derived from the first recorded dementia-specific GP observation record, and therefore all of the sample population had recorded GP observations. However, not all experienced each of the healthcare types. Though nearly all had non-dementia medications (99.4%), just over half had dementia medications prescribed (53.5%). Over four in five of the sample population had A&E attendances (82.3%) and emergency hospital admissions (81.1%), but approximately only two in five had elective hospital admissions (40.3%).

### Multivariable logistic regression: primary and secondary healthcare use

**Figure 2.**
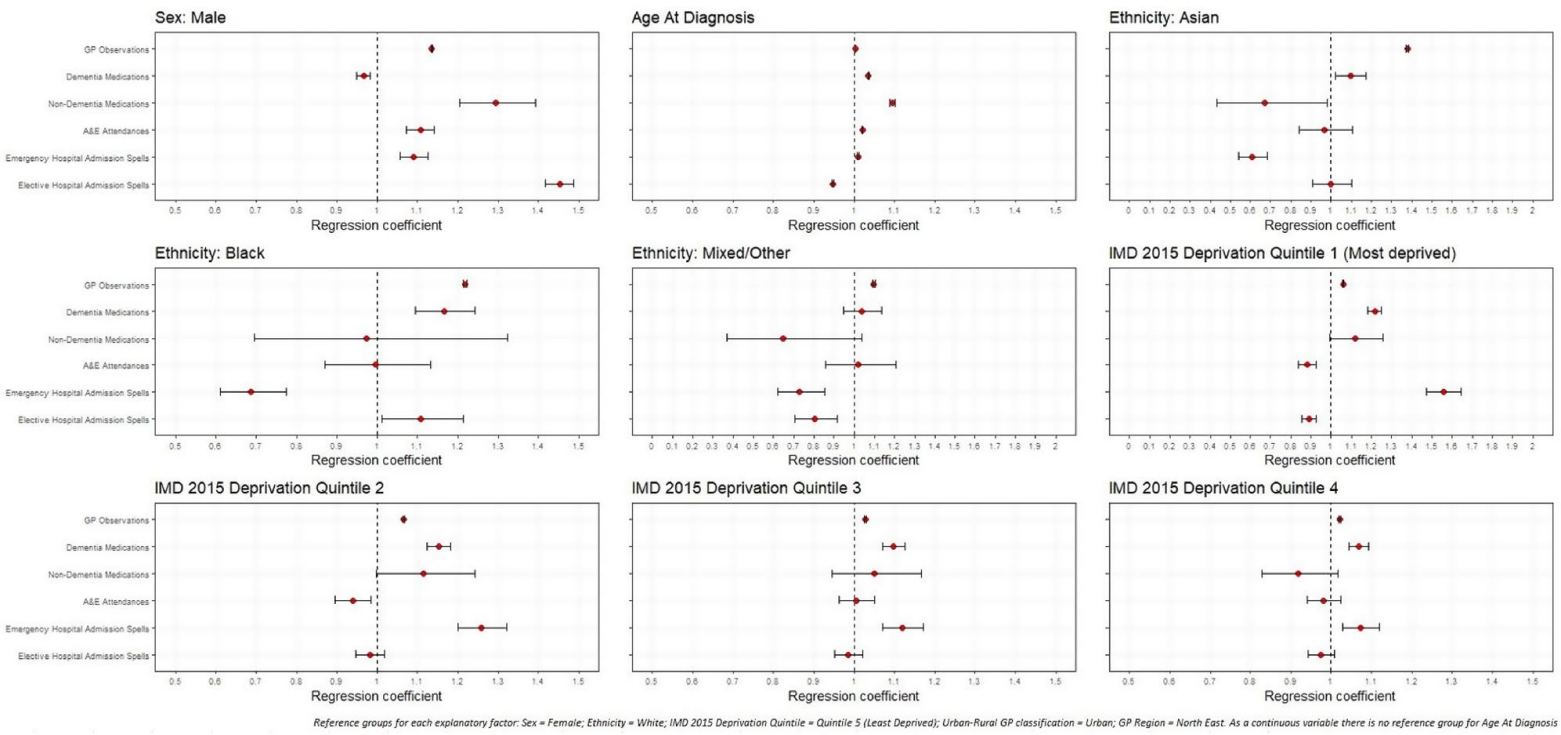
Regression coefficients for healthcare use among later-onset dementia sample population, by demographic and socio-economic factors.

**Figure 3.**
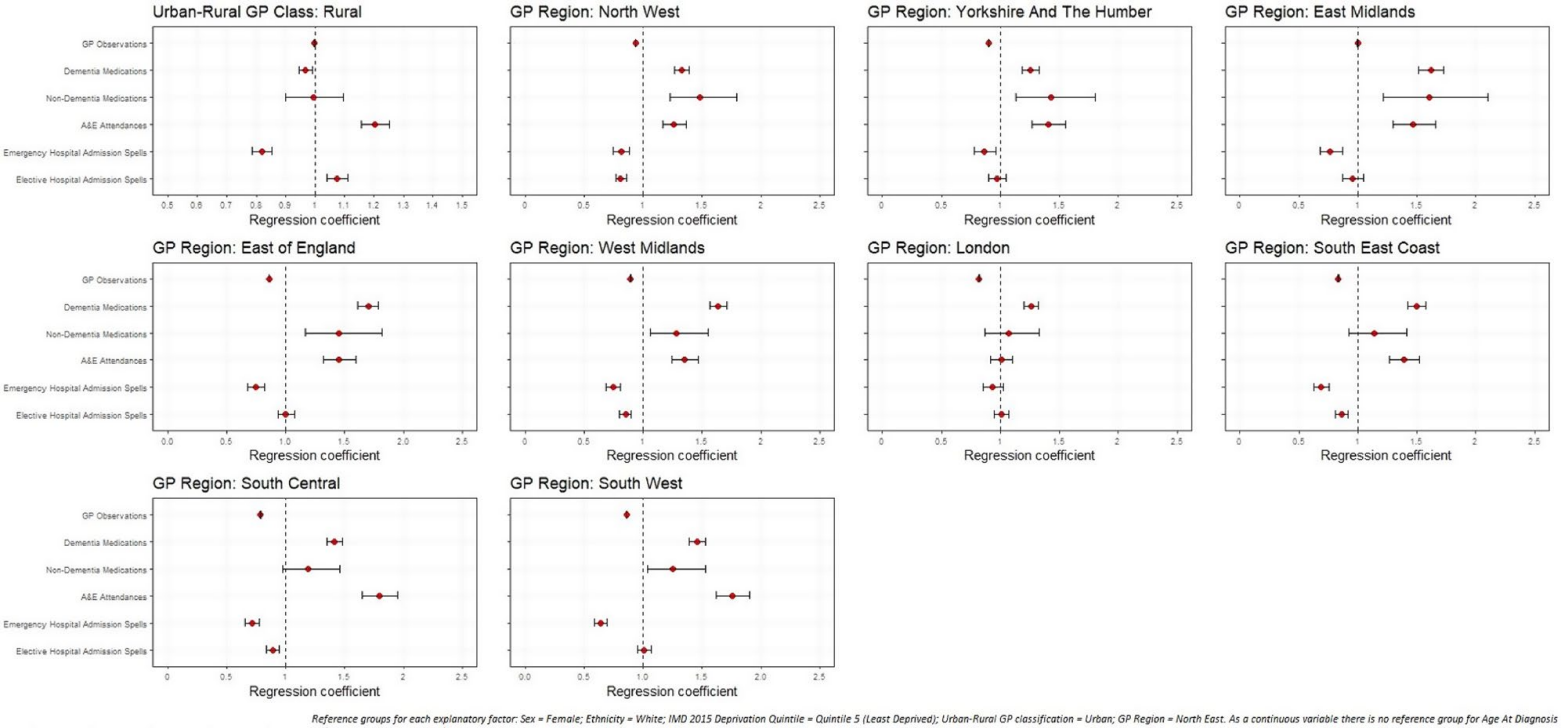
Regression coefficients for healthcare use among later-onset dementia sample population, by spatial factors.

Significant differences in rates of healthcare use were noted by all explanatory factors. Variations were noted among those with early- (Appendices A and B; Page 40-41) and late-onset dementia (Figures 2 and 3), but more so among people living with later-onset dementia.

#### Sex

Compared to women as our reference group, men had significantly more GP observations (early-onset: IRR: 1.077; 1.070–1.084; late-onset: IRR: 1.136; 1.135–1.138) and non-dementia medications (early-onset: IRR: 1.026; 1.019–1.034; late-onset: IRR: 1.295; 1.204–1.392). Men with late-onset dementia had 11% higher odds of attending A&E than women (OR: 1.107; 1.073–1.142). Men were also more likely be admitted to hospital than women, whether as an elective (OR: 1.452; 1.418–1.487) or emergency (OR: 1.090; 1.056–1.125).

#### Age

Increasing age was significantly associated with greater GP observations (early-onset: IRR: 1.002; 1.001–1.002; late-onset: IRR: 1.003; 1.003–1.003) and non-dementia medications (early-onset: IRR: 1.012; 1.011–1.013; late-onset: IRR: 1.095; 1.089–1.101). The youngest (early-onset: IRR: 0.967; 0.956–0.978) and oldest (late-onset: IRR: 1.035; 1.034–1.036) had the most dementia medications. Among those with late-onset, each year increase in age resulted in a 2% greater likelihood of using A&E (OR: 1.020; 1.018–1.022) and emergency hospital admission spells (OR: 1.009; 1.007–1.011), but being less likely to have elective hospital admission spells (OR: 0.947; 0.945–0.949).

#### Ethnicity

Compared to those of White ethnic background, PLWD from Asian (early-onset: IRR: 1.790; 1.762–1.817; late-onset: IRR: 1.377; 1.371–1.383) and Black (early-onset: IRR: 1.213; 1.191–1.237; late-onset: IRR: 1.218; 1.213–1.223) ethnic groups had greater GP observations. Those with late-onset from Black ethnic groups also had significantly greater prescriptions for dementia medication (IRR: 1.167; 1.095–1.243) than those from a White ethnic background, but both people from Black (OR: 0.687; 0.611–0.775) and Asian ethnic groups (OR: 0.608; 0.542–0.683) had a significantly lower likelihood of emergency hospital admission spells. In early-onset dementia, compared to those from White ethnic groups, people from Asian (IRR: 1.607; 1.578–1.637) and Black (IRR: 1.117; 1.092–1.142) ethnic groups had significantly higher rates of non-dementia medications, whereas PLWD from Mixed/Other ethnic groups has significantly fewer (IRR: 0.875; 0.842–0.908).

#### Deprivation

Compared to PLWD from the least deprived quintile (Quintile 5), those in the most deprived quintile (Quintile 1) had significantly higher rates of GP observations (early-onset: IRR: 1.208; 1.195–1.221; late-onset: IRR: 1.059; 1.057–1.061) and, in early-onset had 65% higher rates of non-dementia medications **(**IRR: 1.648; 1.626–1.670) and, in late-onset higher rates of dementia medication prescriptions (IRR: 1.217; 1.184–1.251). In late-onset, compared to the least deprived quintile (Quintile 5), those in the most deprived quintile (Quintile 1) were significantly more likely to be admitted to hospital as an emergency (OR: 1.557; 1.474–1.644), but less likely to attend A&E (OR: 0.880; 0.835–0.926) or have elective hospital admissions (OR: 0.890; 0.856–0.926).

#### Urban–rural GP classification

Among those with early-onset dementia, people with rural GP practices had significantly fewer GP observations (IRR: 0.909; 0.900–0.919) than urban. In later-onset dementia, A&E attendances were more likely among PLWD with rural GPs (OR: 1.204; 1.156–1.253), but emergency hospital admission spells were less likely (OR: 0.820; 0.787–0.855).

#### GP region

Compared to the North East GP region, PLWD registered with GPs in other regions had significantly fewer GP contacts but more non-dementia medications. In late-onset, all GP regions had significantly greater rates of prescriptions for dementia medications than the North East. Among those with late-onset, PLWD in all GP regions apart from London were more likely to attend A&E, but six of the nine regions were significantly less likely to have emergency hospital admissions than the North East.

## Discussion

Our study is one of the first to use large-scale EHR to document social and spatial variation who is accessing and receiving diverse types of healthcare among PLWD. Men and older PLWD were more likely to use primary and emergency secondary healthcare. PLWD from Asian and Black ethnic groups had greater GP contact and in later-onset dementia were less likely to have emergency hospital admissions. Increasing socioeconomic deprivation is also associated with greater GP contact, emergency hospital admissions and medications. PLWD with rural GPs had less GP contact than individuals in urban areas and though they were more likely to attend A&E, were also less likely to have emergency hospital admissions. The North East region had fewest GP contacts, varied medications and likelihood of emergency healthcare use.

We found men had more GP contact, non-dementia medications and both emergency and elective hospital admissions. Higher rates of non-dementia medications among men is a finding consistent with higher levels of severe comorbidities and severe dementia symptoms among men (Gambassi et al., 1999; Lovheim et al., 2009; Lyketsos et al., 1999; Nelis et al., 2019). Men have greater healthcare needs due to greater ill-health (Bertogg and Strauss, 2020; Sharma et al., 2016). Men with dementia also have shorter (Ono et al., 2010), but more frequent, hospital admissions than women and upon hospital discharge are more likely return to be readmitted to hospital (Bartlett et al., 2018; Watson et al., 2020).

This study reported greater use of primary healthcare, and lower risk of emergency hospital admissions, for people with late-onset dementia from ethnic minority backgrounds. The factors impacting healthcare use among PLWD from ethnic minority backgrounds is nuanced. Increased GP contact among these groups may reflect greater need for treatment due to more chronic health conditions (Price et al., 2013; Quinones et al., 2019), as well as primary healthcare being more equitable for ethnic minorities than other forms of healthcare (King’s Fund, 2021). However, our findings emphasise less need for acute healthcare among PLWD from ethnic minority backgrounds. There is lower mortality risk among PLWD from ethnic minority backgrounds (Watson et al., 2021), a finding which may be consistent with younger demographics (insufficiently controlled for in our analysis) and reduced severity of dementia (Parveen & Oyebode, 2018). Existing research highlights the barriers in accessing quality healthcare for PLWD from ethnic minority backgrounds (Cooper et al., 2010; Lin et al., 2020; Mukadam et al., 2011; Pham et al., 2018), but with reduced severity, there is also less frequent contact with healthcare services (Duran-Kirac et al., 2022).

We found that people with late-onset dementia from the most deprived areas had higher GP observations, dementia medications and increased likelihood of using emergency healthcare. Although literature tends to show that PLWD from areas of greater deprivation receive fewer medications for dementia (Cooper et al., 2016; Vohra et al., 2021), our findings emphasise the difficulties in accessing to quality healthcare in more deprived areas. Access to dementia diagnosis and subsequent treatment is more difficult in more deprived areas (Hoang et al., 2021). PLWD from deprived areas are more likely to experience poorer quality primary healthcare (Watson et al., 2020; Wu et al., 2018) and receive a late or unspecified dementia diagnosis that can make effective medicative treatment, where feasible, more difficult (Connolly et al., 2011; Jitlal et al., 2021; Petersen et al., 2021). In this study, although PLWD from the most deprived areas had increased contact with a range of different types of healthcare, this may be indicative of greater and more acute need for treatment of both dementia, and other comorbidities (Browne et al., 2017; Jitlal et al., 2021; Watson et al., 2020).

In addition, we found significant differences in experiences between urban and rural areas, suggesting the importance of spatial factors in determining healthcare experiences. In early-onset, people with dementia registered with rural GPs had less contact with their GP, and those with late-onset had greater likelihood of attending A&E. Health and social care services are more sparse in rural areas (Baird and Wright, 2006; Bauer et al., 2019; Giebel, 2020; National Centre for Rural health and Care, 2022) and PLWD from rural areas are more likely to live with relatives than those in urban areas (Rahman et al., 2020). Sparsity of local services may also mean PLWD registered with rural GPs have a greater reliance on their GP to act as gatekeeper to diagnosis and treatment (Szymczynska et al., 2011). This emphasised reliance on GPs, along with few available services may result in a lack of care management and effective treatment (Bayly et al., 2020; Dal Bello-Haas et al., 2014), which can lead to a greater need for more acute, emergency healthcare, including A&E attendances.

### Limitations

We have included over 120 million records of primary and secondary healthcare contacts for 142,302 people diagnosed with dementia in England. We have identified social and spatial differences in the frequency and likelihood of contact with six different types of healthcare, highlighting variations in potentially avoidable service use, and healthcare use more closely associated with negative health outcomes. There are potential issues with bias and representativeness of the population being studied. Given the nature of dementia and process of diagnosis, it is difficult to pinpoint the exact date of diagnosis in health records. Although there are methods to test for symptoms of dementia, they are not prevalent in primary healthcare, consistently applied, or always appropriate, and there remains a reliance on clinical judgement during healthcare contacts (Chithiramohan et al., 2019; Creavin et al., 2017; Lin et al., 2015). Lack of GP time, confidence in diagnosing or lack of knowledge of dementia in primary care may result in issues around the diagnosis (Phillips et al., 2012). This means fewer PLWD will have an official diagnosis, which impacts some socio-economic groups more than others; our findings may not therefore be reflective of the entire population of PLWD. While we have access to socio-economic and demographic variables to allow adjustment for their influences in analyses, some population groups are under-represented through lack of dementia diagnoses, including people from an ethnic minority background and those living in more deprived areas (Connolly et al., 2011; Pham et al., 2018). This may result in selection bias being introduced in our data, including biasing the associations between our exposures and outcomes (Hindorff et al., 2018; Williams & Cooper, 2019). There is a need to improve data collection, with routine data including more characteristics for PLWD, enabling research to be inclusionary and represent the population being studied. Finally, our analyses are descriptive (i.e. identifying differences by social and spatial factors) rather than interrogating explanations for why these social and spatial variations exist. This is partly a limitation of our data source since we are constrained in what data is provided on electronic health records (both about treatments/outcomes and individual’s contexts). Future research should identify explanatory reasons and pathways for these associations, including the complexity linking our outcomes to measures of inequalities (e.g. provision of informal care, lack of GPs in some areas limiting care received or disentangling whether medications are given based on need or demand). Where possible, these analyses should be extended longitudinally to explore sequences of healthcare trajectories that can consider how healthcare experiences operate holistically rather than independently (as in our analyses).

## Conclusions

Our findings suggest there are wide social and spatial differences in the use of various healthcare services among PLWD. Early identification of dementia, as well as better care management and effective treatment, can help avoid unnecessary healthcare use associated with negative outcomes among PLWD, benefitting not only PLWD, but reducing the costs and pressure on the healthcare system (Banerjee and Wittenberg, 2009; Delgado et al., 2022; Rasmussen and Langerman, 2019). Our findings show the ongoing pressing need for clinical and public health policy aimed at promoting more equitable healthcare experiences among PLWD. This requires implementation of systemic, cultural and social measures to improve the situation for more marginalised groups (Giebel, 2020; Watson et al., 2020). Greater emphasis is required to make quality care easily accessible to people from more remote and deprived areas, and more appropriate to the communities they serve (Duran-Kirac et al., 2022; Giebel, 2020; Nebel et al., 2018). PLWD would benefit from more ubiquitous, effective management and treatment of dementia and comorbidities, in primary and specialist healthcare (Black et al., 2015). Better continuity of primary care, and stronger links between primary and social care, would allow smoother transitions and stability in changing care needs (Delgado et al., 2022).

## Appendix C. Healthcare contact rates per patient year for sample population with early-onset dementia, by healthcare type and explanatory factor

**Table:**

			Healthcare contacts per patient year				
Explanatory factor	Patients	Patient years	GP observations	Dementia medication	Non-dementia medication	A&E attendances	Hospital admission spells
Sex							
Female	2,763	29,670	51.32	3.17	39.15	0.38	0.36
Male	2,447	18,238	75.20	4.28	55.68	0.58	0.64
Age group							
Under 45	104	967	73.16	2.00	49.71	0.61	0.60
45–54	870	6,640	70.46	3.76	50.52	0.63	0.56
55–64	4,236	40,301	58.45	3.60	44.51	0.42	0.45
Ethnicity							
Asian	128	978	139.10	4.65	94.03	0.57	0.62
Black	131	976	86.60	4.16	63.14	0.56	0.96
Mixed/Other	54	419	62.96	1.56	34.18	0.53	0.48
White	4,517	42,824	57.76	3.52	44.90	0.47	0.48
IMD 2015 Quintile							
Quintile 1: Most deprived	1,020	7,764	85.54	5.00	73.44	0.67	0.62
Quintile 2	1,003	15,363	38.41	2.06	30.67	0.29	0.29
Quintile 3	1,073	8,151	69.78	4.04	50.11	0.56	0.63
Quintile 4	1,137	9,073	65.80	3.91	46.81	0.48	0.49
Quintile 5: Least deprived	957	7,398	62.98	4.42	40.00	0.44	0.46
Urban/Rural GP classification							
Urban	4,528	42,674	58.89	3.39	44.98	0.46	0.47
Rural	682	5,234	64.63	5.23	49.25	0.42	0.46
GP Region							
North East	261	1,992	87.23	8.43	90.43	0.68	0.63
North West	1,027	8,107	77.88	4.36	55.95	0.59	0.57
Yorkshire and the Humber	223	1,595	65.60	4.64	60.05	0.70	0.73
East Midlands	139	1,091	80.53	4.07	47.95	0.44	0.48
East of England	264	1,887	73.04	3.56	56.85	0.50	0.59
West Midlands	882	6,720	72.38	3.37	53.01	0.55	0.53
London	622	4,877	78.38	4.86	53.20	0.71	0.69
South Central	714	13,608	25.53	1.73	19.23	0.16	0.19
South East Coast	433	3,236	63.02	2.68	41.09	0.51	0.52
South West	645	4,795	70.62	4.76	57.93	0.46	0.54

## Appendix D. Healthcare contact rates per patient year for sample population with later-onset dementia, by healthcare type and explanatory factor

**Table:**

			Healthcare contacts per patient year				
Explanatory factor	Patients	Patient years	GP observations	Dementia medication	Non-dementia medication	A&E attendances	Hospital admission spells
Sex							
Female	91,270	737,292	51.87	3.22	49.83	0.45	0.43
Male	45,822	392,926	53.24	2.92	42.63	0.44	0.51
Age group							
65–74	20,514	194,397	58.03	3.71	46.57	0.40	0.45
75–84	63,225	548,480	54.65	3.42	49.10	0.45	0.47
85–94	49,067	365,512	46.53	2.48	45.39	0.47	0.45
95+	4,286	21,829	41.12	1.08	41.98	0.49	0.43
Ethnicity							
Asian	1,809	9,885	121.77	5.56	116.17	0.90	0.89
Black	2,115	12,548	102.11	6.47	116.96	0.91	0.98
Mixed/Other	1,026	5,651	91.47	6.02	91.72	0.77	0.80
White	125,101	1,062,758	50.99	3.02	45.98	0.45	0.46
IMD 2015 quintile							
Quintile 1: Most deprived	21,342	202,990	49.29	2.79	46.92	0.47	0.46
Quintile 2	23,920	214,270	50.34	2.91	46.34	0.45	0.44
Quintile 3	27,528	188,153	62.56	3.73	56.68	0.53	0.54
Quintile 4	31,641	224,941	58.90	3.50	52.20	0.48	0.50
Quintile 5: Least deprived	32,405	298,422	44.45	2.82	38.72	0.35	0.38
Urban/rural GP classification							
Urban	117,058	1,025,432	49.70	2.90	44.58	0.44	0.44
Rural	20,034	104,786	78.23	5.21	74.22	0.56	0.64
GP region							
North East	7,167	39,293	92.58	8.69	104.52	0.89	0.81
North West	24,395	131,388	87.13	4.74	69.98	0.76	0.73
Yorkshire and the Humber	5,914	110,878	23.83	1.62	22.04	0.21	0.21
East Midlands	2,881	14,668	92.31	3.43	64.94	0.68	0.72
East of England	7,997	52,389	61.29	3.09	56.91	0.46	0.53
West Midlands	23,887	149,052	67.93	3.16	58.03	0.58	0.60
London	14,203	84,330	77.59	5.75	80.46	0.87	0.78
South Central	18,865	377,910	19.21	1.20	17.04	0.14	0.17
South East Coast	11,619	63,856	72.09	3.52	56.66	0.64	0.62
South West	20,164	106,454	78.29	5.01	78.51	0.56	0.66
